# A Review of Entomopathogenic Nematodes as a Biological Control Agent for Red Palm Weevil, *Rhynchophorus ferrugineus* (Coleoptera: Curculionidae)

**DOI:** 10.3390/insects13030245

**Published:** 2022-02-28

**Authors:** Wan Nurashikin-Khairuddin, Siti Noor Aishikin Abdul-Hamid, Mohammad Saiful Mansor, Izwan Bharudin, Zulkefley Othman, Johari Jalinas

**Affiliations:** 1 Department of Biological Sciences and Biotechnology, Faculty of Science and Technology, Universiti Kebangsaan Malaysia, Bangi 43000, Selangor, Malaysia; p107241@siswa.ukm.edu.my (W.N.-K.); msaifulmansor@ukm.edu.my (M.S.M.); ibb@ukm.edu.my (I.B.); 2 Centre for Insect Systematics, Faculty of Science and Technology, Universiti Kebangsaan Malaysia, Bangi 43600, Selangor, Malaysia; 3 Agrobiodiversity and Environment Research Centre, MARDI Headquarters, Serdang 43400, Selangor, Malaysia; ctaikina@mardi.gov.my; 4 Department of Biomedical Sciences, Faculty of Medicine and Health Sciences, Universiti Putra Malaysia (UPM), Serdang 43400, Selangor, Malaysia; zulkefley_os@upm.edu.my

**Keywords:** *Rhynchophorus ferrugineus*, palm infestation, *Steinernema*, *Heterorhabditis*, symbiotic bacteria, biological control

## Abstract

**Simple Summary:**

The red palm weevil, *Rhynchophorus ferrugineus,* is a significant pest to palm plantations globally and directly impacts economic activities. These weevils’ cryptic attack on palms is inconspicuous until the damage is irreparable. Chemical pesticides were used extensively in plantations to mitigate RPW infestation, and the results were impressive. However, their negative impact on the environment, nontarget organisms, and insecticide resistance is a primary concern. Therefore, alternative preventive and curative solutions based on the natural enemy concept are safer for the environment and more sustainable. This review highlights the use of entomopathogenic nematodes and their symbiotic bacteria as biological control agents against the red palm weevil and storage formulation.

**Abstract:**

*Rhynchophorus ferrugineus* (Olivier) (Coleoptera: Curculionidae) is a severe pest of palm trees worldwide. The development and feeding activities of *R. ferrugineus* larvae inside the trunk damage palm trees. However, the absence of noticeable infestation signs at an early stage contributes to the spread of the attack. Integrated pest management (IPM) has been introduced to control *R. ferrugineus* infestation by implementing various approaches and techniques. The application of chemical pesticides has shown impressive results. However, biological control should be applied as an alternative solution due to adverse environmental impacts and pest resistance issues. One example is the use of entomopathogenic nematodes (EPNs) as biological control agents, which can forage and attack targeted pests without compromising the environment and other nontarget organisms. EPNs and their symbiotic bacteria have a mutualistic interaction that can kill the host within a short period of time. Therefore, this review emphasizes the effectiveness of entomopathogenic nematodes and their symbiotic bacteria against *R. ferrugineus*.

## 1. Introduction

The red palm weevil (RPW), *Rhynchophorus ferrugineus* (Olivier) (Coleoptera: Curculionidae), is the primary pest of date palm (*Phoenix dactylifera)*, canary palm (*Phoenix canariensis*), coconut (*Cocos nucifera*), and other palm trees [1,2,3,4]. Approximately 40 palm species are host to the RPW, with the majority being from the Arecaceae family, with one species each from Agavaceae and Poaceae [5]. Malaysia first recorded three *Rhynchophorus* species, i.e., *R. ferrugineus* in Peninsular Malaysia, Sabah, and Sarawak; *Rhynchophorus vulneratus* (Panzer) in Peninsular Malaysia and Sarawak; and *Rhynchophorus bilineatus* (Montrouzier) in Sarawak only [2,6].

RPW infestation has resulted in additional costs to the ornamental and cultivation palm industries, as it involves eradication and treatment processes [7]. Various preventive or curative approaches are currently being applied to combat RPW infestation on palm trees, including physical and chemical detection with chemical and biological control approaches [8,9]. Entomopathogenic nematodes (EPNs) are one of the biological control agents used in integrated pest management (IPM) programs, as they are lethal obligate parasites and safe to the environment. The role of EPNs is similar to entomopathogenic fungi and bacteria in reducing and replacing the use of chemical insecticides [10,11]. Much research has been conducted to test the pathogenicity of EPNs as potential biological control agents of the RPW and their effectiveness in the field. The efficacy of EPNs against the targeted host is diverse, depending on the species or strain. Therefore, this paper discusses the role and application of EPNs as biological control agents in managing RPW infestation in the field.

## 2. Biology and Distribution of *R. ferrugineus*

The genus *Rhynchophorus* consists of ten species distributed in specific regions [4]. *Rhynchophorus cruentatus* (F.) is found in the coastal areas of South Carolina through to the Florida Keys and west into coastal Texas in the USA [12,13]. *Rhynchophorus palmarum* (L.) is native to Mexico, Central and South America, the Caribbean, and Southern California USA [14,15]. *Rhynchophorus ritcheri* (Wattanapongsiri) is native to Peru, *Rhynchophorus quadrangulus* (Quedenfeld) from West to Central Africa, and *Rhynchophorus phoenicis* (F.) in tropical Africa [16,17,18]. *Rhynchophorus bilineatus* is native to New Guinea and Papua, *Rhynchophorus distinctus* (Wattanapongsiri) in Borneo, *Rhynchophorus lobatus* (Ritsema) in Sumatera, and *R. ferrugineus* in Oriental Asia [4]. *R. vulneratus* is native to Southeast Asia, was discovered, and subsequently eradicated from California [19,20]. *R. ferrugineus* and *R. vulneratus* are classified as synonymous and color morphs of the same species based on cytochrome oxidase gene sequence and morphological characteristics [21]. Based on these data, *R. vulneratus* should also be known as *R. ferrugineus*. However, DNA sequences of mitochondrial COI data [22], with COI and Cytb molecular clock analysis [23], have proved that *R. ferrugineus* and *R. vulneratus* are two different species.

The RPW originated from South and Southeast Asia by infesting coconut palms, *C. nucifera*, and was later detected in the Middle East region [24], where it became the key pest of the date palm (*P. dactylifera).* Eventually, the infestation continued to North Africa [25], and Spain was the first country in Europe to report RPW presence in 1993 [26]. The canary and date palms have been the main factors for the rapid expansion of the RPW throughout the Mediterranean during the last two decades [3,27]. In Southeast Asian countries, RPW infestation is not only limited to coconut (*C. nucifera)* but also other important economic plants such as oil palm (*Elaeis guineensis*)*,* sago palm *(Metroxylon sagu),* and sugar palm (*Arenga pinnata*), as well as ornamental plants such as ribbon palm (*Livistona decipiens)* and Chinese fan palm (*Livistona chinensis)* [6,9,28,29]. The RPW quickly bores of young palm below 20 years. as the trunk is soft and tender [30,31,32].

RPWs have four phases in their life cycle: eggs, larvae, pupae, and adults. Corresponding to several articles, the eggs deposited by adult female RPWs vary, ranging from 180 eggs to 396 eggs [33,34,35,36,37]. Depending on where the attack begins along the palm tree, adult females lay eggs in wounds or openings at the palm crown or leaf scar [35]. The complete life cycle of the RPW varies from 45 days to 180 days [2,3]. Once hatched, larvae will start to feed on the soft tissue. Continuous feeding activities will create tunnels along the stem before the larvae develop into pupae inside an oval-shaped cocoon made of palm fibers [6,8,35].

The duration of the larval development phase, which can range from 24 to 210 days, is influenced by diet and temperature, while the host plant species determines the number of larval instars [9,38]. RPWs tend to have a shorter development period if the environment and host plant are favorable to insects [39]. The plant host species influences the fertility of females RPWs [33]. RPWs are reared in laboratory facilities for their continuous supplies in various research, and their diet mainly comes from abundant and nearby sources. Norzainih et al. [40] successfully reared RPWs in the laboratory by feeding them sugarcane and reported the production of eight larval instars within 80 days. In another study, El-Zogby and Abdel-Hameid [41] demonstrated that a sugarcane diet produced 12 instar larvae in 89 days. Different ambient temperatures are likely to be the cause of these inconsistent results. Furthermore, within a year, RPWs can produce 3–4 generations or more in a single palm tree [42,43].

## 3. Red Palm Weevil Infestation

*R. ferrugineus* infestation on canary palms can be classified into five stages, according to Güerri-Agulló et al. [44]. The first level of early infestation begins with the absence of visual indicators of RPW assault on the palm tree and progresses to the formation of pits and notches in the leaves at the second level. The third and fourth stages are characterized by uneven leaves in the crown, with frond skirting pointing downwards. Finally, the palms die in the last stage.

In oil palm, the RPW infestation level can be classified into the early, intermediate I and II, and final stage. The intermediate stage can be detected as early as the fourth week of the attack with sawdust at the trunk’s base and visible dark sap accompanied by odor from the fermentation process. Consequently, the fronds begin to collapse, and at the final stage, the collapsed fronds turn brown. Early and intermediate infestation can be treated by injecting insecticides into the trunk. The final stage requires the trees to be eradicated by cutting them down according to the guidelines provided [45].

## 4. Control Management of the Red Palm Weevil

Several implementations have been performed to manage RPW infestation, including trapping and monitoring, preventive and curative techniques, and plant quarantine treatments [3,38,46,47]. Preventive and curative treatment should be performed at the early stage of pest infestation to protect the palm from further damage and for recovery of the infested palm [5]. The trapping technique involves using pheromones, with both chemical synthesis and food bait showing significant decreases in RPW infestation. However, synthetic food baits are better than natural food baits since they can attract more weevils and can survive over a longer period [48]. Insecticides based on carbamate, organophosphate, phenylpyrazole, and neonicotinoid are used for preventative and curative treatments [38,47,49]. Early detection of RPW infestation can be performed using a trained dog to detect the foul odor of infested trees [9,16]. Alternatively, acoustic detection can be achieved by detecting the feeding activities of larvae within the stem [27,50,51,52]. Several techniques have been applied to control weevil infestation in coconut plants in Malaysia. These include pheromone traps and chemical and physical controls eradicating the infested palm. The chemical controls involve spraying Cypermethrin on the crown, canopy, and stem of the palm and trunk injection with Methamidophos or Monocrotophos [53].

Management of RPW infestation within the palm trunk is also possible by using biological control agents such as EPNs, as they can penetrate, invade, and kill its prospective pests [54]. Much research has been carried out on the effectiveness of entomopathogenic nematodes (EPNs) against insect pests, especially on the RPW [55]. Besides EPNs, *Metarhizhium anisopliae* and *Beuveria bassiana* are entomopathogenic fungi commonly applied in IPM to kill RPWs in the field [56,57,58]. The pathogenicity of indigenous isolates, *M. anisopliae* strain MET-GRA4 against adult red palm weevils (RPWs), was investigated in vitro with different spore viabilities. The isolates were pathogenic, with 100% mortality 21 days after infection [58].

The only virus found in the RPW is the highly potent cytoplasmic polyhedrosis virus (CPV). The virus was first discovered in Kerala, India, where it infected all stages of the RPW. During the late larva stage, infection resulted in malformed adults and significantly reduced insect populations [59,60]. The efficacy of *Bacillus thuringiensis* (Bt), an entomopathogenic bacterium characterized by its production of insecticidal crystal proteins on larvae and adults of *R. ferrugineus*, was also studied. Infection with *B. thuringiensis* subspecies kurstaki identified from Egyptian larvae successfully controlled the RPW in laboratory conditions [60,61]. A study using commercial bacteria-based biopesticide reported the RPW larval mortality was high (>50%) at the lowest concentration (0.5 mg/mL), and it reached 85% at 2.0 mg/mL [62]. Larvae and adult mortality ranged between 46.86–58.36% and 26.79–39.04%, respectively, after 21 days of exposure to *B. thuringiensis* var. kurstaki [63].

Additionally, microwave heating treatment is another approach used to combat pest issues, such as *R. ferrugineus* infestation, without causing any significant harm to the host plant [64,65,66]. Microwave radiation causes hyperthermia in *R. ferrugineus* adults and larvae. This method is safe for the environment and only causes slight dehydration in palm trees [67]. Several plants, such as the French marigold (*Tagetes patula)* [68], Ceylon (*Cinnamomum zeylanicum)* [69], citronella grass (*Cymbopogon nardus)* [70,71], clove (*Syzygium aromaticum*), and cardamom (*Elettaria cardamomum),* are known for their insecticidal properties and have been proven to effectively kill the RPW [72].

## 5. Biology of Entomopathogenic Nematodes

Nematodes are microscopic, multicellular, and nonsegmented bodies of worms under the phylum Nematoda. Nematodes have adapted to live in various environments and have symbiotic relationships with other organisms [73]. The life cycle consists of the egg stage, four larval stages, and adult stage [74]. The life cycle of EPN is 5–10 days, depending on temperature, bacterial symbiont, and ability to suppress the immunity of the insect host [75,76]. EPNs are widely distributed, but the species varies according to geographic regions and habitats [77,78]. There are 30 families of nematodes associated with insects, plants, and vertebrates [79]. Steinermatidae and Heterorhabditidae are significant families widely used as control agents of insects. Currently, there are two genera in Steinernematidae, *Steinernema* Travassos, 1927 (comprises over 30 species), and *Neosteinernema* Nguyen and Smart, 1994 (one species, *Neosteinernema longicurvicauda*). On the other hand, Heterorhabditidae is solely represented by the genus *Heterorhabditis* Poinar 1976 and one species, *Heterorhabditis bacteriophora* [80,81]. However, Hunt and Nguyen reported that 95 valid species of *Steinernema* and 16 species of *Heterohabditis* were described by the end of 2015 [82].

**Figure 1:**
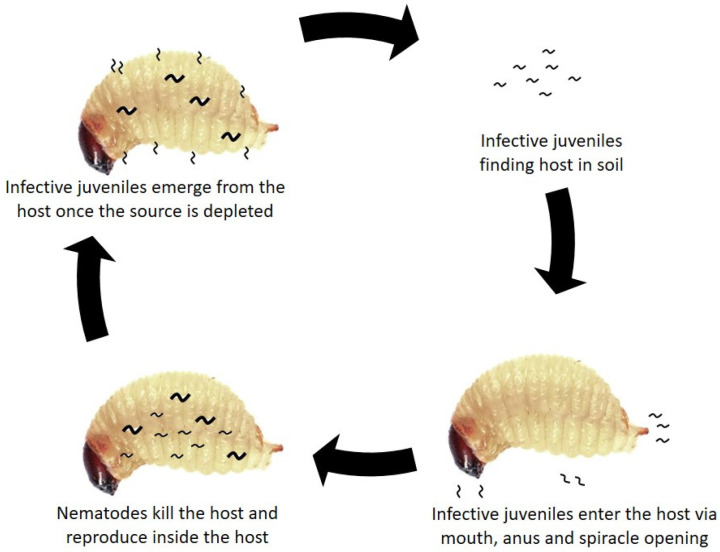
Illustration of invasion of entomopathogenic nematodes on insect host.

A symbiotic Gram-negative bacterium, genus *Xenorhabdus* in *Steinernema* and *Photorhabdus* in *Heterorhabditis*, lives in the modified intestines of infective juvenile (IJ) nematodes [83,84]. When the IJ invade their specific host after entering via the anus, spiracle, and mouth, the symbiotic bacteria released by the IJs in the hemocoel of the target insect multiply and cause the death of the insects within 24–48 h due to septicemia [85,86]. In addition, the symbiotic bacteria produce secondary metabolites that cause cytotoxic activity. It will cause the hemolymph immunodepression of the insect, thus leading to septicemia and death of the host [87]. The secondary metabolites produced by the symbiotic bacteria also inhibit other bacteria, fungus, and protists from developing in the nutrient-rich hemolymph of the dead cadaver, thus providing a suitable condition for the nematodes to reproduce [88,89]. The IJs feed on the multiplied bacteria and digested host tissue until all the sources are depleted and begin to search for a new host [90]. As a result, only infective juvenile (IJ) or dauer larva can survive outside the host [86] (Figure 1). The brown color of the insect cadaver indicates the insect was killed by Steinernematid nematodes, while the red color indicates Heterorhabditid species. The color difference is due to the pigment released by the symbiotic bacteria in the cadaver [80,85].

## 6. Mutualistic Symbiotic Bacteria

In association with *Steinernema* and *Heterorhabditis*, 21 *Xenorhabdus* spp. and 3 *Photorhabdus* spp. have been described, respectively [88,91,92]. The *Steinernema* and *Heterorhabditis* species can be identified through 28S rDNA and ITS regions’ sequencing and PCR-based analysis [88]. Steinernematid reproduces via cross-fertilization between males and females and Heterorhabditid via self-fertile hermaphrodites [90,93,94]. In addition, Heterorhabditid is able to produce hermaphrodites, males, and females in the subsequent generations [95].

The symbiotic bacteria, *Xenorhabdus* and *Photorhabdus*, are essential to the EPN. They kill the insect host in a short period, providing a suitable environment for the EPN to reproduce and produce antibiotics and secondary metabolites that prevent any development of other microorganisms and convert the host tissue into food [96]. In exchange, the EPN provides protection and access to the host’s hemolymph [97]. However, in this bacterium—nematode complex, nematodes are responsible for overcoming the host immunological defense by making lipid or protein [98,99,100]. Thus, the bacterium–nematode complex plays a vital role against insect hosts.

The bacteria *Xenorhabdus* spp. and *Photorhabdus* spp. exist in two phenotypes, primary and secondary [101]. The first-phase bacteria are smaller, 3–4 µm in length, and induce more secretory enzymes, toxins, antibiotics, and protein with the oval or circular shape. The secondary bacteria are larger, 6–7 µm in length, and do not produce enzymes or antibiotics or flat colonies. The primary bacteria only reside in the insect’s body for a few hours after being released by the nematodes before being converted to secondary bacteria. The transformation is believed to adapt to the external environment [102]. Research conducted on *X. stockiae* and *P. luminescens* subsp. *Akhurstii* [88] against *Aedes aegypti* and *Aedes albopictus* has shown that the symbiotic bacteria are malicious against mosquitoes and demonstrated that they could be utilized as biological control agents.

## 7. Application of Entomopathogenic Nematodes as Biological Control Agent

The nematodes use two basic strategies in finding a host: active searching, i.e., cruising or foraging, and the passive method by waiting for the host to contact with the nematodes and ambush [94]. As a result, the EPN can reach its specific host even in a sealed area such as a tree trunk. Major target insects of EPNs in IPM are Coleoptera and Lepidoptera, but it also applies to other orders, such as Thysanoptera, Diptera, Orthoptera, Blattodea, Hymenoptera, and Siphonaptera [81]. Besides that, the application of EPNs against insect pests in the orchard system also contributes to promising results [103]. Shahina et al. [104] conducted a laboratory bioassay of seven EPNs species against all life stages of the RPW, including the eggs. All seven EPNs species killed all stages of the RPW, but the emergence of IJs from the adult RPW was recorded in *Steinernema pakistanense*. However, a single species of EPNs does not have the same virulence towards all stages of the RPW [104,105]. For instance, *Steinernema scapterisci, Steinernema* sp. (SII), *Steinernema abbasi* are virulent to the adult stage, while *Heterorhabditis bacteriophora* is virulent to the larvae stage. However, both stages are susceptible to *Steinernema glaseri*.

## 8. Biological Assay on Pathogenicity of Entomopathogenic Nematodes against *R. ferrugineus*

Various studies on the efficacy of EPNs against the RPW have been conducted, particularly in the Mediterranean, Middle East, and Southern Asia (Table 1). *Steinernema carpocapsae* caused the highest mortality of RPW larvae at 98.9% after eight days of treatment under laboratory conditions, followed by *H. bacteriophora* at 86.9% and *Steinernema feltiae* at 38.9% [55]. The efficacy assessment of EPNs was conducted in the date palm field in the UAE [106], where a local isolate of *Heterorhabditis indicus* appeared to kill larvae, adults, and cocoons, of the RPW successfully within a short period when compared to *Steinernema riobrave* and *S. abbasi*. The RPW population in the date palm field also showed a successful decline after the second treatment within two months. Due to their high pathogenicity against insect hosts [34], the two common EPNs are utilized as biological control agents in IPM are *S. carpocapsae* Weiser and *H. bacteriophora* Poinar [107], as shown in Table 1.

## 9. Formulation

EPNs can be stored and large-scale produced as biopesticides in two ways, in vivo and in vitro [117]. The production of EPNs has enabled at least 13 species of Steinernematids and Heterorhabditids to be commercialized for biological insect control purposes [118]. For small-scale experiments such as laboratory bioassay with low dosage and mortality, the EPN can be inoculated on a dish and absorbent paper before being transferred to White Trap for in vivo harvesting. This method is dependent on environmental conditions that affect yields, such as optimal temperature, proper aeration, and moisture [117]. On the other hand, in vitro methods can offer mass production of the nematodes to fulfill the demanding needs for EPN application in the field. The rearing of nematodes via in vitro methods does not require insects as hosts but through solid or liquid culture [119]. In vitro reliable culture methods are performed by rearing the nematode with respective symbiotic bacteria in a growth medium. In contrast, nematodes are cultured in vitro liquid culture methods after the symbiotic bacteria were introduced in the liquid culture [120]. Next, the mass-produced nematode will be transformed into a new entity or product that practical methods can apply. This process is known as EPN formulation [121] and involves inclusions of carriers, additives, and active ingredients. Various forms and mediums have been used to store and transport EPNs, including aqueous suspension, synthetic sponges, gels, clay, powder, and infected cadaver [122]. These formulations are widely used and commercialized in other countries; for instance, *S. carpocapsae* was commercialized byproduct Sanoplant from Switzerland, Helix from Canada, ORTHO Biosafe USA from the USA, and BASF from Germany [97,122].

## 10. Conclusions

*Rhynchophorus ferrugineus* is indeed a ferocious pest in palm trees worldwide. *R. ferrugineus* infestation creates a substantial economic impact on the plantation and food supply. Many infested trees need to be cut down to prevent dispersion towards other trees. Moreover, farmers and planters have to spend more on preventive and curative treatments to prevent the infestation from spreading. Although chemical treatments are effective, they have numerous drawbacks, including pest resistance, worker’s health, and environmental concerns. Therefore, alternative methods can provide a safer approach yet more effective in overcoming this infestation case of *R. ferrugineus*. Microbial entomopathogens are effective biological control agents since each organism has its approach and strategies for invading its potential host. Entomopathogenic nematodes (EPNs) are proven to be effective biological control agents in managing RPW infestation in various studies. EPNs and their symbiotic bacteria are the significant mutualistic duo that can give promising results in overcoming this infestation issue of *R. ferrugineus*. For a more specific outcome, many approaches can be explored in leveraging the pathogenicity properties of the EPNs and their symbiotic bacteria.

## Figures and Tables

**Table 1:** Summary of the pathogenicity assay on red palm weevil by using the entomopathogenic nematode.

Author	Species	Bioassay	Result	Symbiotic Bacteria	Origin/ Country
[55]	*S. corpocapsae* *S. feltiae* *H. bacteriophora*	Concentrations: 100 IJs each larva and adult RPW: 3rd, 6th, 10th larvae, adult Duration: 12 h duration up to 8 days	Mortality: *S. corpocapsae* 3rd: 96.5%, 6th: 94.7%, 10th: 88.17%, Adult: 3.07% *S. feltiae* 3rd: 38.68%, 6th: 36.35%, 10th: 35.35%, Adult: 0% *H. bacteriophora* 3rd: 85.75%, 6th: 78.15%, 10th: 74.4%, Adult: 0.66%	N/A	Pakistan
[34]	*S. affine* *S. carpocapsae* *S. feltiae* *H. bacteriophora*	RPW: Last instar larvae Concentrations: 500 IJs/ larva Duration: mortality recorded after 7th day	Greatest mortality in *H. bacteriophora* and least in *S. affine*	N/A	Turkey
[108]	*H. bacteriophora* *S. abbasi* *S. anomali* *S. carpocapsae* *S. feltiae* *S. glaseri* *S. riobravae* *Steinernema* sp. *S. ritterai* (EGBS) *S. egyptens* *S. kushidai* *Heterorhabditis* sp.	Concentration: 2000 IJs/mL RPW: 5 weevils in a box (young, medium, full-grown larvae, pupa with cocoon, and adult) Duration: mortality recorded every 2 days for 10 days	Some EPNs showed a preference for certain life stages of weevils. *Steinernema* sp. showed the highest mortality, and *S*. *feltiae* was the least virulent species	N/A	Egypt
[98]	*S. carpocapsae*	The antimicrobial response of RPW larvae on *S. carpocapsae* and *X. nematophila*	Living EPNs and symbionts can suppress the antimicrobial response of the RPW	*X. nematophila*	Netherlands
[105]	*S.scapterisci Steinernema* sp. *S. abbasi* *S. glaseri* *H. bacteriophora*	RPW: Five late instar larvae and an adult. Concentration: (156–2000 IJs/mL) of EPN injected into hemocoel. Duration: 10–13 days	Adults are more resistant than the larva stage. *S.glaseri* and *H.* bacteriophora exhibited high virulence toward the RPW larvae	N/A	Egypt
[106]	*H. indicus* *S. riobrave* *S. abbasi*	Concentration: (50, 100, 200, 400, and 800 IJs) Duration: 60 h and 6 days	The local isolate of *H. indicus* is highly pathogenic towards adult RPWs	N/A	UAE
[99]	*S. carpocapsae*	Immune response of the RPW after infection and post-infection of EPN	The EPN can short-term regulate the phenoloxidase activity for its continuity	N/A	Netherlands
[54]	*S. carpocapsae* *H. bacteriophora*	RPW: Various stages of the RPW (small, medium and large larvae, pupae and adults) Concentration: 50–6000 IJs/0.4 mL water Duration: Mortality recorded after 72 h	Increase size of the host reduces its susceptibility Small larvae—500 IJs Medium larvae—2000/6000 IJs Large larvae—6000 IJs Pupae/adults—2000 IJs	N/A	Germany
[104]	*S. pakistanense* *S. asiaticum* *S. abbasi* *S. siamkayai* *S. feltiae* *H. indica* *H. bacteriophora*	RPW: Eggs, first, third, sixth, final stages larvae, adult Concentration: 50–1500 IJs/mL Period: Mortality was recorded between 24 to 168 h	H. *bacteriophora* and *S. siamkayai* showed the highest mortality of larvae while all EPNs showed similar results in adult RPWs	N/A	Pakistan
[109]	*H. indica* *S. carpocapsae*	Young and grown larvae, adult RPW were infected with EPNs in the laboratory and date palm field	In the lab, the mortality RPWs is from 70% to 100%. In the field, the mortality of adults and larvae is 46% and 60%	N/A	UAE
[110]	*S. riobravae* *S. carpocapsae* *Heterorhabditi* sp.	N/A	All species are virulent to larvae and adult RPWs. LC50 of *S. riobravae* *S. carpocapsae* *Heterorhabditis* sp. were 900, 1100, and 1416 IJs/weevil.	N/A	Egypt
[111]	*S. abbasi* *S. carpocapsae* All *S. carpocapsae* S2 *S. riobravae* *S. feltiae* *S. glaseri* *S. anomali* *Heterorhabditis* sp. IS12 *Heterorhabditis sp.* S1 *H. bacteriophora*	RPW: Larvae, pupae, and adults (lab) 2000 IJs/mL Duration: Mortality was calculated after 7 weeks Field trial: 3000 IJs/mL with 300 mL injected into the infected tree Duration: Mortality was calculated after two weeks of treatment	In the lab, all EPNs were virulent to any RPW stages In the field, 66.67% mortality of larvae was caused by *H. bacteriophora*	N/A	Egypt
[112]	*H. bacteriophora*	RPW: 2nd, 4th, and 6th instar larval of RPW. Method: *Beauveria bassiana* and *Metarhizium anisopliae* combined treatment Larval development was recorded. Duration: Mortality of the larvae were recorded weekly after application	Association of *H. bacteriophora* and *B. bassiana* produced better results, especially in early larvae and decelerated larval development	N/A	Pakistan
[113]	*H. bacteriophora* *H. megidids* *H. carpocapsae* *S. feltiae* *S. glaseri* *S. affine* *S. longicaudum* *S. apuliae* *S. kraussel*	Concentration: 300 IJs in 0.5 mL water RPW: Late instars and adult RPW Duration: Mortality was recorded every 2 days in 10 days	*H. bacteriophora, S. longicaudum,* and *S. carpocapsae* were highly virulent towards larvae and adult RPWs. *S. glaseri* was only highly virulent towards RPW larvae only.	*P. luminescens* subsp. *laumondii* *P. luminescens* *X. nematophila* *X. bovieni* *X. ehlersii* *X. kozodoii*	New Zealand Italy USA Germany
[114]	*S.carpocapsae*	An alternate application of EPNs and Imidacloprid on the canary palm as a preventive treatment	Combination applied treatments were able to reduce the population RPWs	N/A	Spain
[115]	*Steinernema carpocapsae*	Product Biorend^®^ was sprayed onto the canary palm. Nine larvae each palm. Period: Inspection after 14 and 28 days post-infection	Restorative and inhibitory of EPNs were at 80% and 98%, respectively	*X. nematophila*	Spain
[68]	*Heterorhabditis bacteriophora*	Concentration: 300 IJs in 1 mL water RPW: 6th instar larvae and adult Duration: Mortality was recorded until 21 days of exposure in laboratory conditions. Treatment combination: *H. bacteriophora* with *Bacillus thuringiesis* Kurstaki (70 µg g^−1)^ and *H. bacteriophora* with *Beauveria bassiana* (1 × 10^7^ conidia mL^−1^)	Mortality percentage of RPW larvae and adults was 92.40% and 81.29%, respectively Mortality percentage of RPW larvae: 93.35–100% (EPN + Bt-k) and 100% (EPN + *B. bassiana*) Mortality percentage of RPW adult: 81.27–94.24% (EPN + Bt-k) and 100% (EPN + *B. bassiana*)	N/A	Pakistan
[116]	*Heterorhabditis bacteriophora*-HP-88	Laboratory condition: Concentration: 250, 500, 1000, 1500, and 2000 IJs/mL RPW: 4th, 8th, 11th instars larvae and adults Duration: Mortality was recorded 24 h till 9 days post-treatment Field condition: Concentration: 2000 IJs/mL Infested tree: Five infested date palm, *Phoenix dactylifera* injected with IJs. Each tree received approximately 2 L of EPN solution. Duration: Infestation was monitored every week until recovery	Mortality percentage of 4th instar larvae was 100% for all concentrations., while LC_50_ for 8th, 11th, and adults was 435.16 IJs/mL, 1045.34IJs/mL, and 167.90 IJs/mL, respectively No external sign of recovery for three weeks of observations	N/A	Egypt
